# Role of METTL20 in regulating β-oxidation and heat production in mice under fasting or ketogenic conditions

**DOI:** 10.1038/s41598-018-19615-4

**Published:** 2018-01-19

**Authors:** Tadahiro Shimazu, Tamio Furuse, Shabeesh Balan, Ikuko Yamada, Shuzo Okuno, Hiroko Iwanari, Takehiro Suzuki, Takao Hamakubo, Naoshi Dohmae, Takeo Yoshikawa, Shigeharu Wakana, Yoichi Shinkai

**Affiliations:** 1 Cellular Memory Laboratory, RIKEN, 2-1 Hirosawa, Wako, Saitama, 351-0198 Japan; 2 Japan Mouse Clinic, RIKEN BRC, 3-1-1 Koyadai, Tsukuba, Ibaraki, 305-0074 Japan; 3 Laboratory for Molecular Psychiatry, RIKEN Brain Science Institute, Wako, Saitama, 351-0198 Japan; 4 Graduate School of Biostudies, Kyoto University, Kyoto, Kyoto, 606-8507 Japan; 5 Department of Quantitative Biology and Medicine, Research Center for Advanced Science and Technology, The University of Tokyo, 4-6-1 Komaba, Meguro-ku, Tokyo, 153-8904 Japan; 6 Biomolecular Characterization Unit, RIKEN Center for Sustainable Resource Science, Wako, Saitama, 351-0198 Japan

## Abstract

METTL20 is a seven-β-strand methyltransferase that is localised to the mitochondria and tri-methylates the electron transfer flavoprotein (ETF) β subunit (ETFB) at lysines 200 and 203. It has been shown that METTL20 decreases the ability of ETF to extract electrons from medium-chain acyl-coenzyme A (CoA) dehydrogenase (MCAD) and glutaryl-CoA dehydrogenase *in vitro*. METTL20-mediated methylation of ETFB influences the oxygen consumption rate in permeabilised mitochondria, suggesting that METTL20-mediated ETFB methylation may also play a regulatory role in mitochondrial metabolism. In this study, we generated *Mettl20* knockout (KO) mice to uncover the *in vivo* functions of METTL20. The KO mice were viable, and a loss of ETFB methylation was confirmed. *In vitro* enzymatic assays revealed that mitochondrial ETF activity was higher in the KO mice than in wild-type mice, suggesting that the KO mice had higher β-oxidation capacity. Calorimetric analysis showed that the KO mice fed a ketogenic diet had higher oxygen consumption and heat production. A subsequent cold tolerance test conducted after 24 h of fasting indicated that the KO mice had a better ability to maintain their body temperature in cold environments. Thus, METTL20 regulates ETF activity and heat production through lysine methylation when β-oxidation is highly activated.

## Introduction

Protein post-translational modifications (PTMs) are involved in diverse cellular processes such as gene expression, signal transduction, and intracellular interactions. Lysine methylation in histones has been extensively studied for more than a decade; it is known to play important roles in gene expression, replication, and genome stability^1–3^. Although hundreds of non-histone lysine methylation sites have been reported, the enzymes responsible for methylation of these lysines and the physiological functions of these PTMs remain largely unknown.

There are approximately 40 MTase genes that may participate in mitochondrial functioning because the encoded proteins contain mitochondria-targeting signal sequences^4^. Nonetheless, only a small number of these genes has been characterised so far. METTL20 was the first mitochondrial lysine MTase (KMT) to be discovered (by two independent groups)^5,6^. METTL20 specifically methylates the β subunit (ETFB) of electron transfer flavoprotein (ETF), which acts as a mobile carrier of electrons from several flavin adenine dinucleotide–containing dehydrogenases to ETF: quinone oxidoreductase. The two METTL20 methylation sites in ETFB, i.e. K200 and K203, are located in the proximity of the recognition loop responsible for the interaction between ETF and medium-chain acyl-coenzyme A (CoA) dehydrogenase (MCAD)^7^; it is believed to be involved in interactions with other ETF-dependent dehydrogenases. Accordingly, it has been shown that METTL20 decreases the ability of ETF to extract electrons from MCAD and glutaryl-CoA dehydrogenase *in vitro*^6^. Moreover, METTL20-mediated methylation of ETFB influences the oxygen consumption rate in permeabilised mitochondria that are supplied with palmitoyl-L-carnitine^5^, suggesting that METTL20-mediated ETFB methylation may play a regulatory role in mitochondrial metabolism.

Several approaches have been developed to probe lysine-methylated proteins specifically. One such approach involves antibodies specific to methylated lysine^8,9^. The second approach takes advantage of methylated lysine-binding domains. It has been reported that an engineered version of the triple malignant-brain-tumour domain region, i.e. L3MBTL1, can serve as a probe for the detection and enrichment of proteins containing mono- and di-methylated lysine residue(s)^10,11^. Another approach is to use cofactor analogues for labelling, detection, and pull-down via copper-catalysed azide-alkyne cycloaddition, also known as click chemistry, to enrich PTM targets. Synthetic co-factors for lysine methylation of several SET domain KMTs have been reported, for example, SUV39H2^12^, G9a/GLP^12,13^, ESET^14^, Set7/9^13^, and a seven-β-strand MTase, METTL10^15^.

In the present study, we performed a proteomic substrate screening for METTL20 in mitochondria of cultured cells. We found that ETFB is a major substrate; this result is consistent with other findings^5,6^. To explore the biological function of METTL20-mediated ETFB methylation, we created *Mettl20* knockout (KO) mice and characterised phenotypes related to their metabolism.

## Results

### ETFB is a major substrate for METTL20 in the mitochondria, and its catalytic activity is regulated by lysine methylation

**Figure 1:**
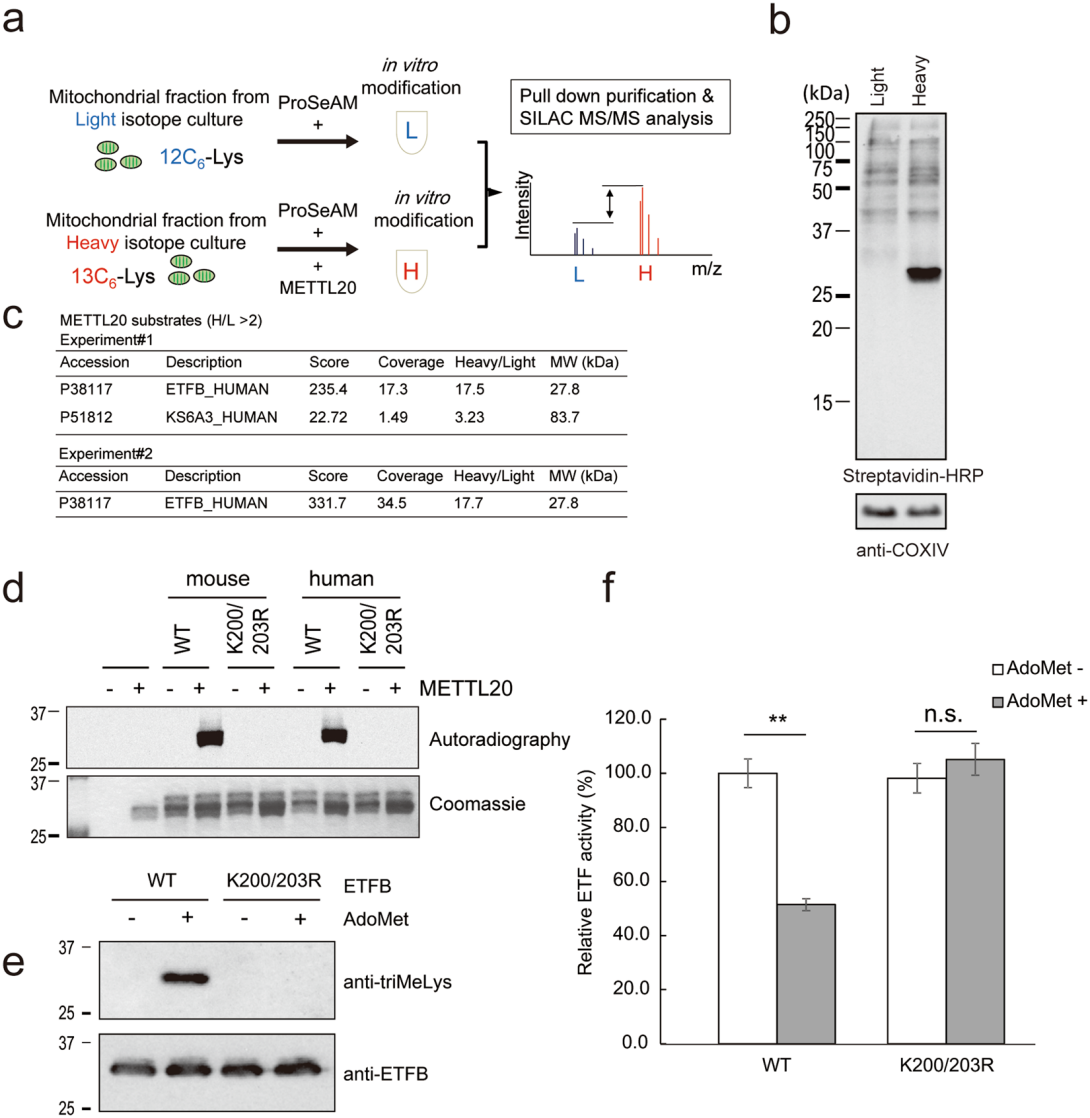
ETFB methylation is conserved in human and mouse, which regulates the catalytic activity. (**a**) A schematic drawing of screening for METTL20 substrates. Cells were cultured in either light isotope labeled Lys containing medium (L) or heavy isotope labeled Lys containing medium (H). The mitochondrial lysates were reacted with ProSeAM with (H) or without (L) His-METTL20. After the *in vitro* modification, biotin tags were introduced to the modified residues via click reaction. Then the two samples were mixed together, and biotinylated proteins were pull-down with Streptavidin beads. After Lys-C digestion, peptides were analyzed by LC-MS/MS. (**b**) Confirmation of ProSeAM labeling. After the ProSeAM mediated alkylation and biotinylation via click reaction, small aliquot of the protein samples were analyzed by SDS-PAGE followed by western blotting with Streptavidin-HRP and anti-COXIV antibody as a loading control. (**c**) METTL20 substrates identified in the screening. Summary of two independent experiments were shown. Note that only ETFB was the protein identified in both cases. (**d**) Recombinant ETF complex (ETFA WT/ ETFB WT or K200/203R) and His-METTL20 were incubated with 14C-labeled AdoMet for 2 h at 30 °C. The autoradiography was detected with the image analyzer BAS-5000. (**e**) 4 µg of the ETF complex were mixed with 4 µg of METTL20 in the presence or absence of AdoMet and incubated at 30 °C for 3 h. After the reaction, they were mixed on ice with 8 µg of His-MCAD, 50 µM octanoyl-CoA and 70 µM DCPIP. Initial rate of the reduction of DCPIP was calculated from OD600 measured at 30 °C for every 30 sec for 10 min with a microplate reader (SpectraMax 190, Molecular Devices). Average of three independent experiments, n = 3 for each experiments, mean ± SEM. Student’s T-test **p < 0.01. (**f**) Small aliquot of the ETF used in A were separated with SDS-PAGE, and their methylation were detected with anti-triMeLys antibodies (top) and anti-ETFB antibodies (bottom, as loading control).

Recently, a mitochondrial MTase, METTL20, was identified as an ETFB MTase^5,6^. We also attempted proteomic identification of METTL20 substrates in mitochondria. A proteomic analysis using a synthetic S-adenosylmethionine (AdoMet) analogue, ProSeAM^12,15^, has been applied to identify the substrate (Fig. 1a). HEK293T cells were cultured in DMEM with 10% of dialyzed foetal calf serum (FCS) containing either light isotopic ^12^C_6_-Lys or heavy ^13^C_6_-Lys. Each mitochondrial fraction was incubated with ProSeAM^12,13,15^ in the absence or presence of His-METTL20. After biotinylation of ProSeAM-labelled proteins, a small part (5%) of the protein was subjected to western blotting with streptavidin conjugated with horseradish peroxidase (HRP; Fig. 1b). The samples were combined and mixed, and subsequent proteomic analysis revealed that ETFB was a major mitochondrial substrate for this enzyme (Fig. 1c).

We confirmed that ETFB was modified by ProSeAM (Supplementary Fig. S2a) and labelled with ^14^C-AdoMet (Supplementary Fig. S2b) in the presence of METTL20 *in vitro*. Because histones contain many methylated lysine residues, we also tested recombinant histone H3 (hereafter: H3) in an experiment and demonstrated that METTL20 can methylate both ETFB and H3 *in vitro*. It should be noted that because histones are nuclear proteins, they are possibly not a physiological target of METTL20, which is localised to mitochondria. Nevertheless, it is still possible that METTL20 has one or more additional mitochondrial substrates aside from ETFB. We also confirmed that the methylation site in ETFB is exactly the same as in other reports, i.e., K200 and K203 (Supplementary Fig. S2c–f)^5,6^. We then checked if this methylation is conserved between humans and mice. As shown in Fig. 1d, methylation of murine ETFB was abolished in K200/203R mutant, indicating that methylation of ETF is well conserved between these two species.

ETF functions as an electron transfer protein for acyl-CoA dehydrogenases. Given that the methylation of ETFB inhibits the catalytic activity^5,6^, we also checked the mouse ETF catalytic activity *in vitro*. The recombinant ETF complex was incubated with METTL20, with or without AdoMet for 3 h, after which the ETF complex was mixed with recombinant MCAD, octanoyl-CoA, and 2,6-Dichloroindophenol (DCPIP) as described previously^16,17^. The methylation status of ETFB after the methylation reaction was confirmed (Fig. 1e). As depicted in Fig. 1f, methylated ETF showed more reduced activity (51% ± 2%) than unmethylated ETF did (100% ± 5%). It can be inferred that either the KR mutant did not affect catalytic activity (98% ± 5%) or the methylation reaction did not influence its activity (105% ± 6%). These results confirmed that methylation of lysine in ETFB by METTL20 inhibits mouse ETF catalytic activity, in line with the results from other reports^5,6,18^.

### Creation of *Mettl20* KO mice by CRISPR/Cas9 genome editing

**Figure 2:**
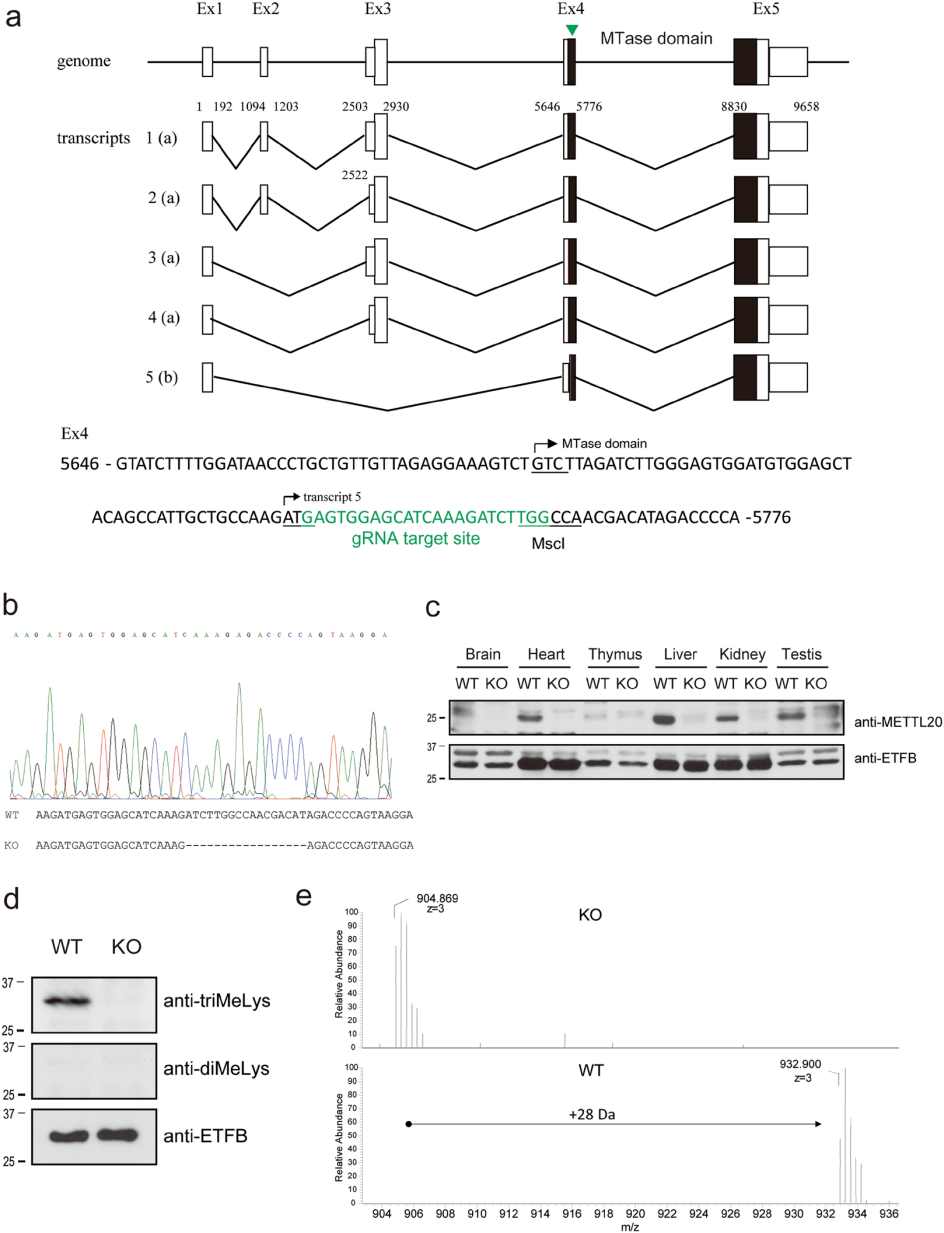
Generation of Mettl20 knockout mouse. (**a**) Schematics of mouse *Mettl20* gene. *Mettl20* has five transcripts. Transcript 1 to 4 encode same longer isoform (**a**) but possess different 5′ UTR, whereas transcript 5 encodes shorter isoform (**b**), which start translation in exon4. A guide RNA was designed against the Ex4. Small box; transcript including UTRs, large box; coding region, filled box; MTase domain, arrow head; gRNA target site. (**b**) DNA sequence of the *Mettl20* KO mice. The KO mice lost 17 base pairs around the gRNA target site which resulted in a frame-shift mutation of Mettl20. (**c**) Protein expression of METTL20 in WT and KO mice. Several tissues were collected from WT and KO mice, and protein expression of mMETTL20 or ETFB were measured with a western blotting with anti-Mettl20 antibodies (top) or anti-ETFB antibodies (bottom). (**d**) Methylation of endogenous ETFB in the mice liver. Mitochondria were fractionated from WT or *Mettl20* KO mice liver, and endogenous ETFB were immunoprecipitated with anti-ETFB antibodies. Precipitated proteins were separated with SDS-PAGE, and their methylation were detected with anti-triMeLys antibodies (top), anti-diMeLys antibodies (mixture of c4930 and c5123) (middle), and anti-ETFB antibodies (bottom, as loading control). (**e**) nanoLC-MS analysis of endogenous ETFB. Immunoprecipitated ETFB were separated with SDS-PAGE, digested with Asp-N, and methylation of peptides containing K200/203 were analyzed with nanoLC-MS (Q-Exactive, Thermo Fisher Scientific). Detected triply charged peptide ion were m/z = 904.869 (z = 3), which corresponds to un-modified EPRYATLPNIMKAKKKKIEVVKAG peptide in KO mice and m/z = 932.900 (z = 3), which corresponds to the peptide carrying 6 methyl groups in WT.

To understand the physiological function of METTL20 *in vivo*, *Mettl20* KO mice were generated via the CRISPR/Cas9 genome editing technology^19,20^. Guide RNAs were designed against exon 4, which encodes the MTase domain of METTL20 (Fig. 2a). The guide RNA sequence contains the MscI site, which is convenient for genotyping. A *Mettl20* KO mouse carries a 17 bp deletion, which results in a frameshift mutation in the MTase domain (Fig. 2b). Expression of mMETTL20 in several organs of WT and KO mice was examined by western blotting with an anti-METTL20 antibody. As shown in Fig. 2c, mMETTL20 protein expression levels were undetectable in all the tissues tested in KO mice but were the highest in the WT liver. Because proteins METTL20 and ETFB were highly expressed in the mouse liver, which is an important organ for fatty acid metabolism, we checked the ETFB methylation level in the liver of WT and KO mice. ETFB in the liver was immunoprecipitated with an anti-ETFB antibody, and methylation was detected with an anti-di-methyl lysine antibody (diMeLys) or anti-tri-methyl lysine antibody (triMeLys, Fig. 2d). Tri-methylation of ETFB was not detected in KO mice, suggesting that mMETTL20 was crucial for the tri-methylation. Of note, di-methylation of ETF was detected in neither WT nor KO mice. To verify this result, the ETFB immunoprecipitated from WT and *Mettl20* KO liver samples was subjected to liquid chromatography-mass spectrometry (LC-MS). As shown in Fig. 2e, the K200 and K203-containing ETF peptide had an additional molecular weight (MW) of 28 Da in WT mice, in agreement with the MW observed after 2× trimethylation. In contrast, this additional MW was completely lost in KO mice. These observations implied that K200 and K203 were fully tri-methylated in ETFB of WT mice but remained completely unmethylated in *Mettl20* KO mice.

### *Mettl20* KO mice show increased ETF activity, oxygen consumption, and heat production when fed a ketogenic diet (KD) or when fasted

**Figure 3:**
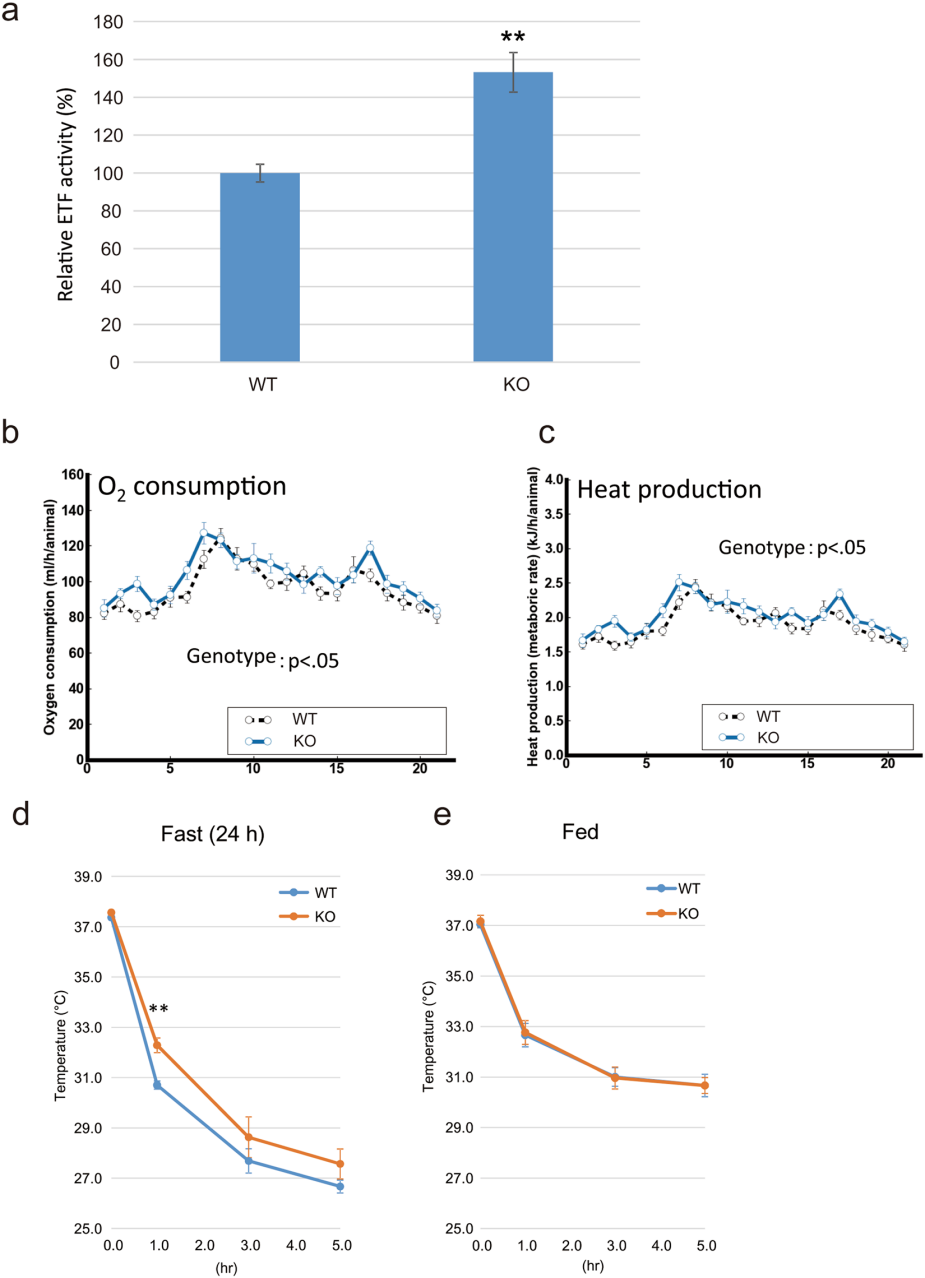
The *Mettl20* KO mice had higher O2 consumptions and heat production under KD and showed cold tolerance under fasting. (**a**) ETF activity in mitochondrial lysate. 300 µg of the mitochondrial fraction from mice liver were sonicated, and the supernatant was mixed with 3 µg recombinant MCAD, 80 µM octanoyl-CoA and 70 µM DCPIP. Initial rate of the reduction of DCPIP was calculated from OD600 measured at 30 °C for every 30 sec for 10 min with a microplate reader (SpectraMax 190, Molecular Devices). Average of three independent experiments, n >= 3 for each experiments, mean ± SEM. Student’s T-test **p < 0.01. (**b**) Oxygen consumption fed with KD. ANOVA, effect of genotype, F (1, 18) = 5.0746, P < 0.04; effect of time increment, F (20, 360) = 13.5184, P < 0.0001; interaction between genotype and time increment, F (20, 360) = 1.053, P > 0.39. Horizontal axis indicates time increment (h). Error bars represent the S.E.M. N = 10 male mice for each genotype at 12 weeks of age. (**c**) Heat production fed with KD. ANOVA, effect of genotype, F (1, 18) = 4.8976, P < 0.05; effect of time increment, F (20, 360) = 13.2229, P < 0.0001; interaction between genotype and time increment, F (20, 360) = 1.061, P > 0.38. Horizontal axis indicates time increment (h). Error bars represent the S.E.M. N = 10 male mice for each genotype at 12 weeks of age. (**d**) Cold tolerance test under fasting and (**e**), Fed. WT and *Mettl20* KO mice were either fed on ND (**e**) or fasted for 24 h (**d**) before the experiment. Mice in the same cage per conditions/genotype were transferred to cold room (4 °C), and measured their core body temperatures after 1 h, 3 h and 5 h. n = 5, mean ± SEM. Student’s T-test **p < 0.01.

We measured ETF activity in *Mettl20* KO mice as reported elsewhere^17^, with a slight modification. Briefly, the mitochondrial fractions from the WT or KO murine liver were sonicated, and the supernatant was mixed with recombinant MCAD, octanoyl-CoA, and DCPIP. As presented in Fig. 3a, the ETF activity in *Mettl20* KO mouse mitochondria was ~1.5-fold greater than that in the WT. This result is consistent with that of *in vitro* experiments, suggesting that methylation of ETFB inhibited ETF activity (Fig. 1f) because ETFB in the WT mitochondria was fully methylated (Fig. 2d and e).

The growth of *Mettl20* KO mice was normal and indistinguishable from that of the WT when these mice were fed a normal diet (ND) or KD. Additionally, the differences in the body weight and several parameters such as results of liver function testing (LDH, AST, ALT, and GGT), renal function testing (TP, ALB, UN, CRE), lipid metabolism analyses (T-Bil, HDL-C, LDL-C, T-CHO), glucose, IPGTT, and serum adipocytokine tests were statistically insignificantly different between WT and KO mice (data not shown).

To examine the possibility that *Mettl20* KO mice were better at generating energy from fat, a calorimetric analysis was performed on the mice fed the ND or KD, because the KO mice showed increased ETF activity (Fig. 3a). Both strains of mice had similar food intake and motor activity throughout the experiments (data not shown). As shown in Fig. 3b and c, the *Mettl20* KO mice fed the KD exhibited increased oxygen consumption and heat production (p < 0.05), whereas these parameters were unchanged when these mice were fed the ND (Supplementary Fig. S3).

Next, we checked heat production in a cold environment (Fig. 3d and e). The mice were either fed or fasted for 24 h before the experiment, given that fatty acid metabolism is known to be elevated during fasting. The cold tolerance test indicated that *Mettl20* KO mice had a better ability to maintain their body temperature than WT mice did during fasting (Fig. 3d, KO: 32.3 °C ± 0.3 °C vs. WT: 30.7 °C ± 0.2 °C, p < 0.01 after a 1 h cold test). Again, no difference was observed during feeding (Fig. 3e). These results indicated that mMETTL20 is involved in the regulation of β-oxidation under ketogenic or fasting conditions, where fatty acid metabolism is known to be highly accelerated.

### Metabolomic analysis of serum fatty acids in *Mettl20* KO mice

**Figure 4:**
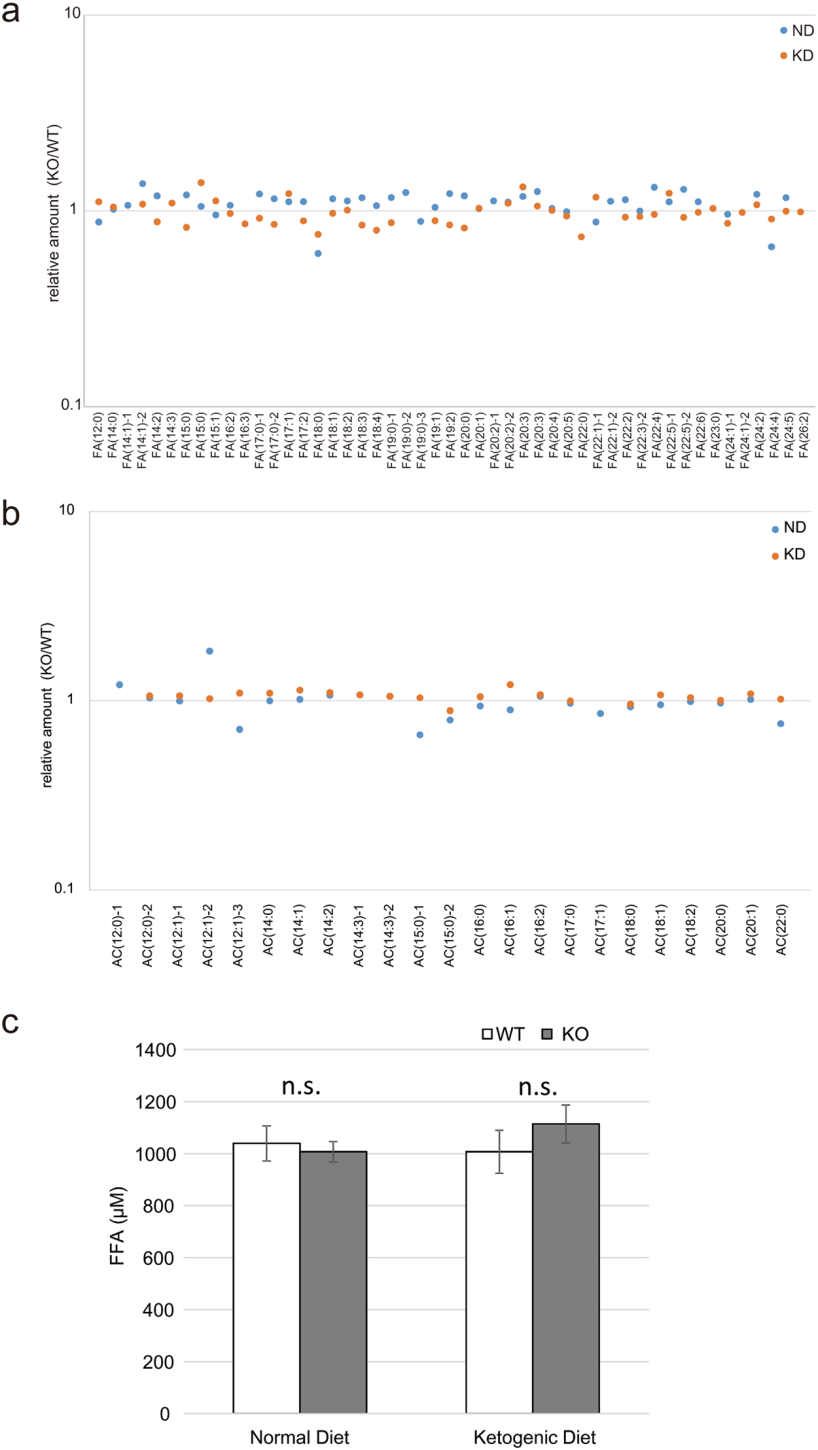
Metabolomic analysis of the *Mettl20* KO mice fed with ND or KD. WT and *Mettl20* KO male mice (n = 10 each condition) were fed with ND or KD from 4 wks old to 26 wks old. Then, mice were sacrificed and the serum were pooled in one tube per condition. Metabolites were extracted and analyzed with LC-TOF MS. (**a**) Serum fatty acids level. (**b**) Serum acyl carnitine level. All metabolomic data were collected as relative amount (KO/WT). (**c**) Blood samples were collected from the WT and *Mettl20* KO male mice (n = 10 each condition) under ad libitum feeding. Concentration of serum free fatty acids (FFA) were measured. Data were presented as mean ± SEM, n = 10 per condition.

Because *Mettl20* KO mice showed accelerated ETF activity and heat production during ketosis or fasting, it is possible that serum fatty acid contents are different between the WT and KO mice. To test this possibility, a metabolomic assay was performed to quantify fatty acids and acyl carnitines in WT or KO mouse serum samples. Overall, the levels of 177 metabolites detected in the assay were not significantly different between the two groups (<2-fold difference, data not shown). Moreover, levels of free fatty acids (FFAs) and acyl carnitine were similar between the two strains of mice (Fig. 4a and b). To confirm these results, we measured the total FFA concentration in serum (Fig. 4c). Again, there was no difference in the serum FFA concentration between the WT and KO mice.

### *METTL20* is tolerant to loss-of-function (LoF) mutations

**Figure 5:**
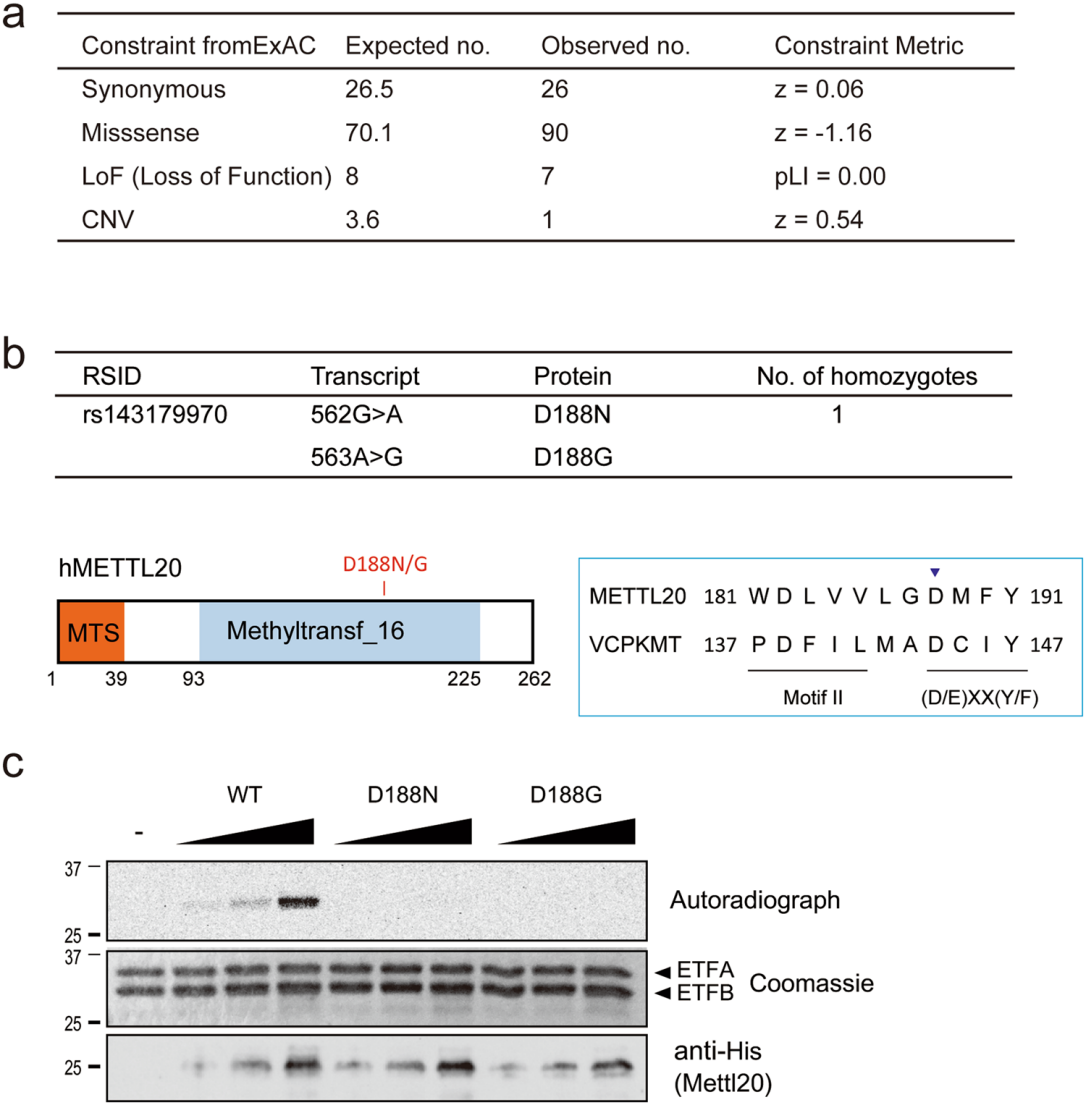
*Mettl20* is tolerant to loss of function (LoF) mutation. (**a**) Genomic variation within the exon of *Mettl20* was screened with ExAC Browser. (**b**) Missense mutations in METTL20 MTase domain found in the ExAC. D188N was found as a homozygous mutation (top). Amino acid sequence of METTL20 and VCPKMT, a Methyltransferase Family 16 with hallmark (D/E)XX(Y/F) motif located downstream of Motif II (bottom). (**c**) Catalytic activity of METTL20 D188N and D188G mutants. Recombinant ETF complex and increased amount of His-METTL20 were incubated with 14C-labeled AdoMet for 2 h at 30 °C. Proteins were separated with SDS-PAGE and stained with Coomassie blue (middle, loading control), the autoradiography was detected with the image analyzer BAS-5000 (top). Loading amount of METTL20 was confirmed with a western blotting with anti-His antibody (bottom).

The *METTL20/ETFBKMT* homozygous loss-of-function mutation has been identified in an Icelandic population^21^, suggesting that this enzyme is not essential for viability of humans^18^. To further address the biological significance of genetic variants of *METTL20* in the human population, we searched for genomic mutations within the exons of *METTL20* in the ExAC Browser, which stores exome DNA sequence data for 60,706 individuals with diverse ancestries^22^. As shown in Fig. 5a, *METTL20* was classified as a gene tolerant to LoF (if pLI = 0.00 or pLI < 0.01, then the gene is thought to be tolerant to LoF^22^). In addition to the frameshift mutation that was reported in the Icelandic population^21^, individuals exist that are homozygous for certain missense mutations in METTL20 (ref.^22^ and Supplementary Table S1). Among them, the c.562G > A mutation (rs143179970) induces the D188N substitution in Motif Post II of the METTL20 MTase domain (Fig. 5b), which is crucial for the catalytic activity of seven-β-strand MTases such as VCPKMT^23^. To evaluate the enzymatic activity of the corresponding mutant protein, recombinant METTL20 D188N and another mutant (D188G), which is also present as a heterozygous mutation in the ExAC database, were expressed in bacteria; an MTase activity assay was conducted *in vitro* (Fig. 5c). These mutant enzymes could not methylate ETFB, implying that the loss of catalytic activity by METTL20 does not cause an obvious disadvantage for survival of humans. These findings confirmed that *METTL20* is a gene tolerant to LoF.

## Discussion

Approximately 40 MTase genes may participate in mitochondrial functions^4^; however, only a few of these ‘mitochondrial’ MTase genes have been characterised so far. METTL20 has been identified as an ETFB MTase^5,6^. We also identified ETFB as a METTL20 substrate by a unique method that involves the synthetic co-factor ProSeAM^12,15^. During the screening, ETFB was found to be the sole candidate for METTL20 (Fig. 1c), suggesting that ETFB is a major target of METTL20 in mitochondrial lysates. In the *in vitro* experiments, METTL20 methylated ETFB at K200 and K203, and we could detect peptides carrying up to five methyl groups, but not fully methylated ones (Supplementary Fig. S2f). This observation is interesting because ETFB is fully methylated in the mouse liver (Fig. 2d,e). It is possible that other proteins or co-factors are required for full methylation of ETFB *in vivo*.

Given that methylation sites are located around the recognition loop, which is required for the interaction with MCAD, it is probable that methylation of mouse ETFB affects its catalytic activity (Fig. 1f), and this notion is consistent with the results from other reports^5,6^. In this study, we generated *Mettl20* KO mice (Fig. 2) and demonstrated that methylation of ETF was lost in KO mice. The catalytic activity of ETF was higher in KO mice (Fig. 3a), suggesting that mMETTL20 has a regulatory role in fatty acid oxidation. Accordingly, *Mettl20* KO mice also had a higher oxygen consumption rate and heat production when they consumed the KD (Fig. 3b and c). Furthermore, the KO mice showed greater cold tolerance than the WT did during fasting although there was no difference during *ad libitum* feeding, where glucose metabolism is known to be a primary energy source (Fig. 3d and e). From these results, we can conclude that β-oxidation activity is regulated by the methylation of ETFB by METTL20, when fatty acids serve as a primary energy source, i.e. β-oxidation is highly activated. It should be noted that serum fatty acid concentrations were not affected by the *Mettl20* KO (Fig. 4), pointing to the existence of a compensatory mechanism ensuring lipid homeostasis. Further research is needed to reveal such a compensatory mechanism.

The fact that *Mettl20* KO mice grew to be normal and undistinguishable from WT mice (data not shown) implies that this gene is not essential. To test whether this is true for humans, the ExAC database was searched for genomic variations in *Mettl20* exons. The analysis revealed that *METTL20* is classified as a gene tolerant to LoF (Fig. 5a). The identified homozygous missense mutation (D118N) completely eliminated the METTL20 catalytic activity (Fig. 5c). These results further indicate that METTL20-mediated protein methylation is not essential in humans but more likely modulates energy production during fasting or under other metabolic conditions where β-oxidation is up-regulated. One possible benefit of this regulation is that too much energy production during fasting is avoided. This mechanism may help animals survive long-term starvation. Besides, it is likely that methylation of ETFB dynamically changes according to the metabolic environment, which, in turn, regulates metabolism adaptability. In both cases, further experiments are needed to test these possibilities.

## Materials and Methods

### Animals

All the experiments involving mice were carried out according to protocols approved by the Animal Experiment Committee of the RIKEN Brain Science Institute. The animals were maintained in a 12 h light/dark cycle with *ad libitum* access to food and water. The temperature and humidity were maintained at 22–23 °C and 50–60%, respectively. Animal health was checked by the animal facility staff five times per week. The mice were subdivided into two groups and fed a standard chow (ND) containing 5% fat and 25% protein (CLEA Japan, Inc., CE-2) or a KD containing 91% fat and 9% protein (Harlan Teklad, TD96355) from the age of 4 weeks to 26 weeks.

### Plasmids

Full-length cDNA corresponding to *Mettl20* (NCBI ID: AK132096), *Etfb* (AK150293), *Mcad* (AK146444), *METTL20* (NM_001135863), *ETFA* (NM_000126), and *ETFB* (NM_001985) were obtained either by PCR from a template plasmid, as a clone from the HeLa cDNA library, or as a clone from the Fantom3 clone collection^24^. The cDNAs of METTL20 and MCAD were cloned into the pET19b vector to generate N-terminally His-tagged constructs (pET19b-METTL20, pET19b-METTL20ΔMTS, and pET19b-MCADΔMTS). For tandem expression of the ETFA–ETFB complex, mouse *Etfb* cDNA was cloned at BamHI/NotI sites into MCS1, and human *ETFA* cDNA was cloned at NdeI/XhoI sites into MCS2 of the pETDuet-1 vector (resulting in pETDuet-1-His-ETFB/ETFA). In addition, the cDNAs for METTL20 and ETFB were cloned into the pcDNA3 vector with a C-terminal FLAG tag (pcDNA3-cFLAG) to generate mammalian expression constructs (pcDNA3-METTL20-cFLAG and pcDNA3-ETFB-cFLAG). The QuikChange Site-Directed Mutagenesis Kit (Agilent Technologies, Inc., Santa Clara, CA USA) was used to generate mutant plasmids.

### Antibodies

The following antibodies were used: an anti-ETFB antibody (N-16, Santa Cruz Biotechnology, USA, and PA5-22005, Thermo Fisher Scientific, USA), anti-α-tubulin antibody (clone B-5-1-2, Sigma-Aldrich, St. Louis, MO, USA), anti-FLAG M2 antibody (Sigma-Aldrich), anti-COXIV antibody (Cell Signaling Technology, USA), and anti-6xHis antibody (Wako). Monoclonal anti-di-methylated lysine antibodies (diMeLys) c4930 and c5123 were generated as described previously^25,26^ by means of a chemically lysine-di-methylated baculovirus as an antigen. Their specificity for lysine-di-methylation was confirmed by an ELISA as described elsewhere^27^ (Supplementary Fig. S1). A polyclonal rabbit anti-tri-methyl-lysine antibody (triMeLys) was purchased from Abcam, USA (#ab76118). To generate the anti-METTL20 antibody, 2 mg of recombinant His-METTL20ΔMTS was employed as an antigen to immunise rabbits. The antiserum was collected after three doses of immunisation. Specificity of the immunoreaction was confirmed with an ELISA. Prior to experiments, the antiserum was purified on a column with immobilised METTL20 (antigen).

### Purification of recombinant proteins

BL21 (pLysS) strains were transformed with pET19b plasmids, and the bacteria were cultured in the 2xYT medium with ampicillin (100 µg/ml) and 0.2 mM isopropyl β-D-1-thiogalactopyranoside (IPTG) for 18 h at 16 °C. The cells were pelleted and lysed with 1× PBS containing 0.5% of NP-40 by sonication with a Branson Sonifier (S-250D, Branson Ultrasonics Corp., CT, USA) for 5 min on ice. The lysates were centrifuged at 15,000× *g* for 10 min, and the supernatants were incubated with Ni-NTA Agarose (Qiagen, Valencia, CA, USA) for 1 h at 4 °C with gentle agitation. The agarose beads were washed five times with wash buffer (50 mM Tris-HCl pH 7.4, 25 mM imidazole) and then the bound proteins were eluted with elution buffer (50 mM Tris-HCl, pH 7.4, 250 mM imidazole). The purified proteins were dialyzed against 1× PBS containing 10% of glycerol, and their concentration was measured with the Bradford Protein Assay Kit (BioRad Laboratories, Hercules, CA, USA).

### Stable isotopic labelling of cells and mitochondrial purification

For METTL20 target screening, HEK293T cells were cultured for at least six cell doublings in DMEM supplemented with 10% of dialysed FCS containing either a light (^12^C_6_) or stable heavy (^13^C_6_) isotope in lysine after processing with the SILAC labelling kit (Pierce, cat. # 89983). The mitochondrial fractions of each cell type were purified as described previously^28^. Briefly, cell pellets were resuspended in SEM buffer (10 mM 4-[2-hydroxyethyl]-1-piperazineethanesulfonic acid [HEPES]-KOH pH 7.5, 0.2 M mannitol, 50 mM sucrose, 10 mM KCl, 1 mM EDTA, 0.2 mM phenylmethylsulfonyl fluoride), and homogenised by means of the Dounce tissue grinder (2 ml, Pestle A, Fisher Scientific, USA). After that, the supernatants were centrifuged at 700× *g* for 10 min to separate nuclear pellets from the supernatants containing cytosolic materials and mitochondria. The supernatants were again centrifuged at 700× *g* to remove residual pellets, and then at 7,000× *g* for 10 min to separate the mitochondrial pellet from the cytosol-enriched supernatant. The mitochondrial pellets were next washed twice with SEM buffer, and the resulting mitochondrial fractions were stored at −80 °C.

### ProSeAM labelling

Labelling of mitochondrial proteins with ProSeAM was carried out by a previously described procedure^15^, with a slight modification. Briefly, 10 ul of lysis buffer (50 mM Tris-HCl pH 8.0, 50 mM KCl, 10% glycerol, 0.1% of Tween 20) containing 150 μg of either light-isotope–labelled mitochondria (^12^C_6_-Mito) or heavy-isotope–labelled mitochondria (^13^C_6_-Mito) was incubated with 0.4 mM ProSeAM, with (^13^C-Mito) or without (^12^C-Mito) His-METTL20 (10 μg) (protein:enzyme at 15:1) in 50 ul of MTase reaction buffer (50 mM Tris-HCl pH 8.0) at 20 °C for 2 h. The reaction was stopped by adding four volumes of ice-cold acetone. The reaction tube was centrifuged at 15,000× *g* for 5 min, and the precipitate was washed once with ice-cold acetone. The pellet was dissolved in 58.5 µl of 1× PBS containing 0.2% of SDS, after which 15 µl of 5× click reaction buffer and 1.5 µl of 10 mM Azide-PEG4-Biotin (Click Chemistry Tools) were added; the reaction mixture was incubated for 60 min at room temperature. The click reaction was stopped with four volumes of ice-cold acetone. The pellet was dissolved in 75 µl of binding buffer (1× PBS, 0.1% of Tween-20, 2% of SDS, 20 mM dithiothreitol) and sonicated for 10 s. ^12^C-Mito and ^13^C-Mito samples were mixed in a tube; 450 µl of IP buffer (Tris Buffered Saline (TBS) with 0.1% Tween 20) containing 3 mg of Dynabeads M-280 Streptavidin (Life Technologies Japan Ltd., Minato-ku, Tokyo, Japan) was added into the tube, and it was incubated for 30 min at room temperature (the final SDS concentration in the reaction was 0.5%). The protein-bound beads were washed thrice with wash buffer (1× PBS, 0.1% of Tween 20, 0.5% of SDS) and twice with 100 mM ammonium bicarbonate buffer, and were analysed by western blotting or MS.

### Quantitative tandem MS (MS/MS) analysis for target protein identification

Acetonitrile (1/10 volume) and dithiothreitol (20 mM) were added to protein-bound Dynabeads in 100 mM ammonium bicarbonate buffer, and the mixture was incubated for 30 min at 56 °C. Then, iodoacetamide was added, and the mixture was incubated for 30 min at 37 °C in the dark. Next, the protein samples were digested with 0.5 µg of Lys-C (Promega). The protein fragments were applied to a liquid chromatograph (EASY-nLC 1000; Thermo Fisher Scientific, Odense, Denmark) coupled to a Q Exactive Hybrid Quadrupole-Orbitrap Mass Spectrometer (Thermo Fisher Scientific, Inc., San Jose, CA, USA), with a nanospray ion source in positive mode. The peptides derived from the protein fragments were separated on a NANO-HPLC C18 capillary column (0.075 mm inner diameter × 150 mm length, 3 mm particle size; Nikkyo Technos, Tokyo, Japan). Mobile phase A was composed of water with 0.1% of formic acid, and mobile phase B consisted of acetonitrile with 0.1% of formic acid. Two different slopes were used for gradient elution for 120 min at a flow rate of 300 nl/min: 0–30% of phase B during 100 min and 30–65% of phase B for 20 min. The mass spectrometer was operated in top-10 data-dependent scan mode. The operating parameters of the mass spectrometer were as follows: spray voltage, 2.3 kV; capillary temperature, 275 °C; mass-to-charge (m/z) ratio, 350–1800; normalised collision energy, 28%. Raw data were acquired via the Xcalibur software (Thermo Fisher Scientific). The MS and MS/MS data were subjected to searches in the Swiss-Prot database by means of Proteome Discoverer 1.4 (Thermo Fisher Scientific) in the MASCOT search engine software version No. 2.4.1 (Matrix Science, London, United Kingdom). The search parameters were as follows: enzyme, Lys-C; quantitation, SILAC K (+6); static modifications, carbamidomethyl (Cys); dynamic modifications, oxidation (Met); precursor mass tolerance, ± 6 ppm; fragment mass tolerance, ± 20 mDa; and maximum missed cleavages, 1. The proteins were considered identified when their false discovery rate was less than 1%. For substrate identification, proteins manifesting at least a 2-fold increase in levels in two independent experiments were defined as positive hit proteins.

### An *in vitro* MTase assay

Recombinant histone H3.1 was purchased from NEB. One microgram of the substrate was incubated in 10 ul of 1× Reaction buffer (50 mM Tris-HCl pH 8.0) with His-METTL20 (1 µg) and ^14^C-labelled AdoMet (0.01 µCi, Perkin Elmer) from 2 h to overnight at 30 °C. The reaction was stopped by adding Laemmli SDS sample buffer. Proteins were resolved by SDS-PAGE in a 12.5% acrylamide gel. An imaging plate (Fuji-Film) was exposed to the dry gel for 48 h, and the autoradiograph was obtained on a BAS-5000 Image analyser (Fuji-Film).

### An *in vitro* ETF activity assay

ETF enzymatic activity was determined by a previously described DCPIP dye assay^16,17^. The recombinant WT and mutant ETF complex were prepared with the pETDuet-1-His-ETFB/ETFA. Four micrograms of the ETF complex was mixed with 4 µg of METTL20 with or without AdoMet and incubated at 30 °C for 3 h in 20 ul of MTase reaction buffer. After that, 4 µg of the ETF complex was mixed on ice with 8 µg of His-MCAD, 50 µM octanoyl-CoA, and 70 µM DCPIP in potassium phosphate buffer (pH 7.6) containing 0.2 mM N-Ethylmaleimide (NEM). For the mitochondrial ETF activity assay, 300 µg of the mitochondrial fraction from the murine liver was sonicated, and the supernatant was mixed with 3 µg of recombinant MCAD, 80 µM octanoyl-CoA, and 70 µM DCPIP in potassium phosphate buffer (pH 7.6) containing 0.2 mM NEM. The initial rate of reduction of DCPIP was calculated from optical density at 600 nm measured at 30 °C every 30 s for 10 min on a microplate reader (SpectraMax 190, Molecular Devices). Data were obtained in triplicate for each biological sample.

### Creation of *Mettl20* KO mice

Guide RNAs that target exon 4 of mouse *Mettl20* were screened with CRISPR design tools (http://crispr.mit.edu/). The T7 promoter sequence 5′-TTAATACGACTCACTATAGG-3′ and the 20-mer Mettl20 guide RNA sequence 5′-GAGTGGAGCATCAAAGATCT-3′ were cloned into the AflII site of the gRNA_Cloning Vector, a gift from George Church (Addgene #41824)^19^. Oligos Insert-F (5′-TTTCTTGGCTTTATATATCTTAATACGACTCACTATAGGAGTGGAGCATCAAAGATCT-3′) and Insert-R (5′-GACTAGCCTTATTTTAACTTGCTATTTCTAGCTCTAAAACAGATCTTTGATGCTCCACTC-3′) were annealed and extended to construct a 100-bp double-stranded DNA fragment using Phusion polymerase (New England Biolabs, Japan). The gRNA_Cloning Vector was linearised with AflII and the inserts were incorporated via Gibson assembly (NEB, Japan, E2611S). To prepare *hCas9* mRNA and *Mettl20* guide RNAs, an *in vitro* transcription reaction kit with T7 RNA polymerase (mMESSAGE mMACHINE T7 Ultra Kit, Life Technologies) was used; the transcribed RNAs were purified with the MEGAclear Kit (Life Technologies). The quality of the guide RNA was checked by an *in vitro* Cas9 cleavage assay with recombinant hCas9 nuclease (NEB) and a 1 kb PCR product around the guide RNA target site. The *Cas9* mRNA and guide RNA were injected into fertilised eggs (mouse strain C57BL/6J), and the mutated *Mettl20* gene versions were screened by PCR after MscI digestion, because the MscI site TGGCCA is located immediately after the guide RNA target site. One in 11 heterozygous (+/−) males carried the expected 17 bp deletion. The heterozygous mouse was crossed with a WT C57BL/6J mouse, and the male (+/−) and female (+/−) mice were crossed to obtain *Mettl20* KO (−/−) mice and WT (+/+) mice.

### Calorimetric analysis

We conducted this analysis according to the standard operating procedure for the indirect calorimetry test (IMPC_CAL_001, http://www.mousephenotype.org/impress/protocol/86) of the International Mouse Phenotyping Consortium^29,30^. Briefly, mice were acclimated to the test room (in a 12 h light–dark cycle; lights on at 8:00 h) and the calorimetry cage 24 h before testing. Into the calorimetry cage, a single mouse was placed; food and water were made available *ad libitum* during acclimation and calorimetric analysis. The measurements were started 5 h before lights were switched off for 21 h. Input and output levels of gases oxygen (O_2_) and carbon dioxide (CO_2_) were measured by an Oxymax indirect calorimetry system (Columbus Instruments, Columbus, OH). The changes in the CO_2_ and O_2_ levels between the input and output were used to compute O_2_ consumption (VO_2_; ml/h per animal) and CO_2_ production (VCO_2_; ml/h per animal).

The respiratory exchange ratio (RER), calorific value (CV), and heat production (KJ/h per animal) were computed from VCO_2_ and VO_2_ as follows: RER = VCO_2_/VO_2_, CV = 3.815 + (1.232 × RER); heat production (KJ/h per animal) = 4.184 × CV × VO_2_. Water intake was measured by the Food and Water Intake Monitor (FWI-16M, O’Hara Co., Ltd., Tokyo, Japan). Locomotor activity of the mice was quantified by the Infrared XY beam activity system for mice (IA-16M, O’Hara Co., Ltd.).

### Cold tolerance test

This test was performed as described previously^31^, with a slight modification. WT and *Mettl20* KO mice were either fed the ND or fasted for 24 h before the experiment. Mice in the same cage in terms of conditions and genotype were transferred to the cold room (4 °C), and their core body temperature was measured with a BAT-7001H Thermometer (Physitemp Instruments, Inc.) after 1, 3, and 5 h.

### Metabolomic analysis

WT and *Mettl20* KO male mice (n = 10 for each condition) were fed the ND containing 5% of fat and 25% of protein (CLEA Japan, Inc., CE-2) or the KD containing 91% fat and 9% protein (Harlan Teklad, TD96355) from the age of 4 weeks to 26 weeks. Twenty-six-week-old mice were killed, and the serum samples of mice from each group were pooled and kept at −80 °C until measurements. All LC-TOF MS analyses were conducted at Human Metabolome Technologies Inc., Tsuruoka, Japan. Briefly, 500 μl of pooled serum was mixed with 6 μM internal standards in 1.5 ml of the mixture of 1% formic acid and 99% acetonitrile. Samples were centrifuged at 2,300× *g* and 4 °C for 5 min, and the supernatants were collected and filtered through a solid-phase extraction column. Flow-through fractions were dried and dissolved in 100 μl of 50% 2-propanol. The samples were applied to a liquid chromatograph (Agilent 1200 series RRLC system SL, Agilent Technologies, Inc.) coupled to an Agilent LC/MSD TOF Mass Spectrometer (Agilent Technologies, Inc), with electrospray ionisation in both positive and negative modes. The samples were separated on an ODS column (2 mm inner diameter × 50 mm length, 2 μm particle size). Mobile phase A comprised water with 0.1% of formic acid, and mobile phase B comprised 65% of isopropanol, 30% of acetonitrile, 5% of H_2_O with 0.1% of formic acid, and 2 mM HCOONH_4_. Three slopes were used for gradient elution for 20 min at a flow rate of 300 μl/min: 0–1% of phase B for 0.5 min, 1–100% of phase B during 13.5 min, and then 100% of phase B for 20 min. The settings of the mass spectrometer were as follows: nebuliser pressure, 40 psi; dry gas flow, 10 l/min; dry gas temperature, 350 °C; capillary voltage, 3500 V; scan range, m/z 100–1700. The obtained data were subjected to searches in the HMT database by means of MasterHands ver.2.17.1.11 (ref.^32^) (Keio University). The overall data processing flow consisted of the following steps: noise filtering, baseline removal, migration time correction, peak detection, and integration of the peak area from a 0.02 m/z–wide slice of the electropherograms.

### Fatty acid measurement

WT and *Mettl20* KO male mice (n = 10 for each condition) were fed the ND or KD from the age of 4 weeks to 23 weeks. The blood samples were collected from the orbital sinus during *ad libitum* feeding. Concentrations of serum FFAs were analysed with a kit (Abcam, cat. # ab65341). The obtained data were presented as mean ± SEM, n = 10 per condition.

### ExAC database analysis

The ExAC Browser was searched for the *METTL20* gene mutation^22^ (constraint metrics: for synonymous and missense variants, a signed Z score calculating the deviation of observed counts from the expected number is indicated in ExAC analysis). Positive Z scores indicated increased constraint (intolerance to variation), and therefore the gene had fewer variants than expected. Negative Z scores were given to genes that had more variants than expected.

Regarding LoF, the closer pLI is to 1.0, the more LoF-intolerant the gene is expected to be. If pLI ≥ 0.9, then the set of genes is extremely LoF-intolerant. Among the 106 loss-of-function and missense variants in ExAC (as of September 19, 2017), a D188N mutant (RSID: rs143179970) was found to be a homozygous mutation (Supplementary Table S1).

## Electronic supplementary material

Supplementary Figures
