# Supplementary material for: Role of METTL20 in regulating β-oxidation and heat production in mice under fasting or ketogenic conditions
Source: Sci Rep. 2018 Jan 19;8:1179. doi: 10.1038/s41598-018-19615-4 (PMC5775328; doi:10.1038/s41598-018-19615-4)
Supplement: Supplementary file 1 — Supplementary Figures [file 41598_2018_19615_MOESM1_ESM.pdf]

Supplementary Information

Role of METTL20 in regulating beta-oxidation and heat production in mice under fasting or ketogenic conditions

Tadahiro Shimazu[1], Tamio Furuse[2], Shabeesh Balan[3], Ikuko Yamada[2], Shuzo Okuno[4], Hiroko Iwanari[5], Takehiro Suzuki[6], Takao Hamakubo[5], Naoshi Dohmae[6], Takeo Yoshikawa[3], Shigeharu Wakana[2], Yoichi Shinkai[1§]

[1]Cellular Memory Laboratory, RIKEN, 2-1 Hirosawa, Wako, Saitama 351-0198, Japan.

[2]Japan Mouse Clinic, RIKEN BRC, 3-1-1 Koyadai, Tsukuba, Ibaraki 305-0074, Japan.

[3]Laboratory for Molecular Psychiatry, RIKEN Brain Science Institute, Wako, Saitama 351-0198, Japan. [4]Graduate School of Biostudies, Kyoto University, Kyoto, Kyoto 606-8507, Japan. [5]Department of Quantitative Biology and Medicine, Research Center for Advanced Science and Technology, The University of Tokyo, 4-6-1 Komaba, Meguro-ku, Tokyo 153-8904, Japan.

[6]Biomolecular Characterization Unit, Center for Sustainable Resource Science, RIKEN, Wako, Saitama 351-0198, Japan

Table S1

Homozygote mutation in the ExAC database.

Chrom	Position	RSID	Reference	Alternate	Consequence	Protein Consequence	Transcript Consequence	Annotation	Allele Count	Allele Number	Number of Homozygotes	Allele Frequency
12	31814952	rs138402033	G	A	p.Arg22Gln	p.Arg22Gln	c.65G>A	missense	31	121408	1	2.55E-04
12	31815167	rs61736289	G	T	p.Ala94Ser	p.Ala94Ser	c.280G>T	missense	4521	121018	102	3.74E-02
12	31815202	rs138229541	G	A	c.314+1G>A		c.314+1G>A	splice donor	465	119920	6	3.88E-03
12	31820653	.	T	G	p.Ile173Ser	p.Ile173Ser	c.518T>G	missense	5	121186	1	4.13E-05
12	31820697	rs143179970	G	A	p.Asp188Asn	p.Asp188Asn	c.562G>A	missense	15	121324	1	1.24E-04
12	31820752	.	G	A	p.Cys206Tyr	p.Cys206Tyr	c.617G>A	missense	10	121384	1	8.24E-05
12	31820796	rs151164668	C	T	p.Arg221Trp	p.Arg221Trp	c.661C>T	missense	38	121398	1	3.13E-04
12	31820856	rs115438422	C	T	p.Leu241Phe	p.Leu241Phe	c.721C>T	missense	381	121384	5	3.14E-03

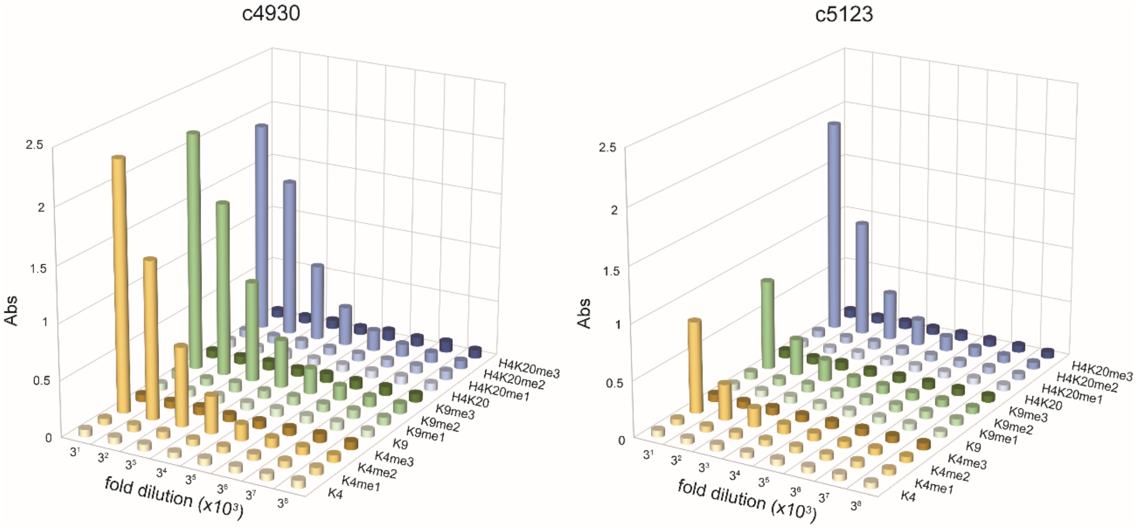

Figure S1. Validation of monoclonal anti-diMeLys antibodies, c4930 and c5123

The specificity of two monoclonal antibodies (c4930 and c5123) was analyzed by ELISA using histone H3 and H4 peptides (H3K4: ARTKQTARKSTGC, H3K9: ARTKQTARKSTGGKAPRKQC, H4K20: GKGGAKRHRKVLRDNIQGIC) containing different modifications. Methylated K is underlined. Microtiter plates coated with the peptides (indicated on the right) were incubated with 3-fold dilutions of each antibody (starting from 1:3000 dilution). After incubation with peroxidase-conjugated secondary antibody and washing, the colorimetric signal of tetramethylbenzidine was detected by measuring the absorbance at 405 nm (Abs) using a plate reader.

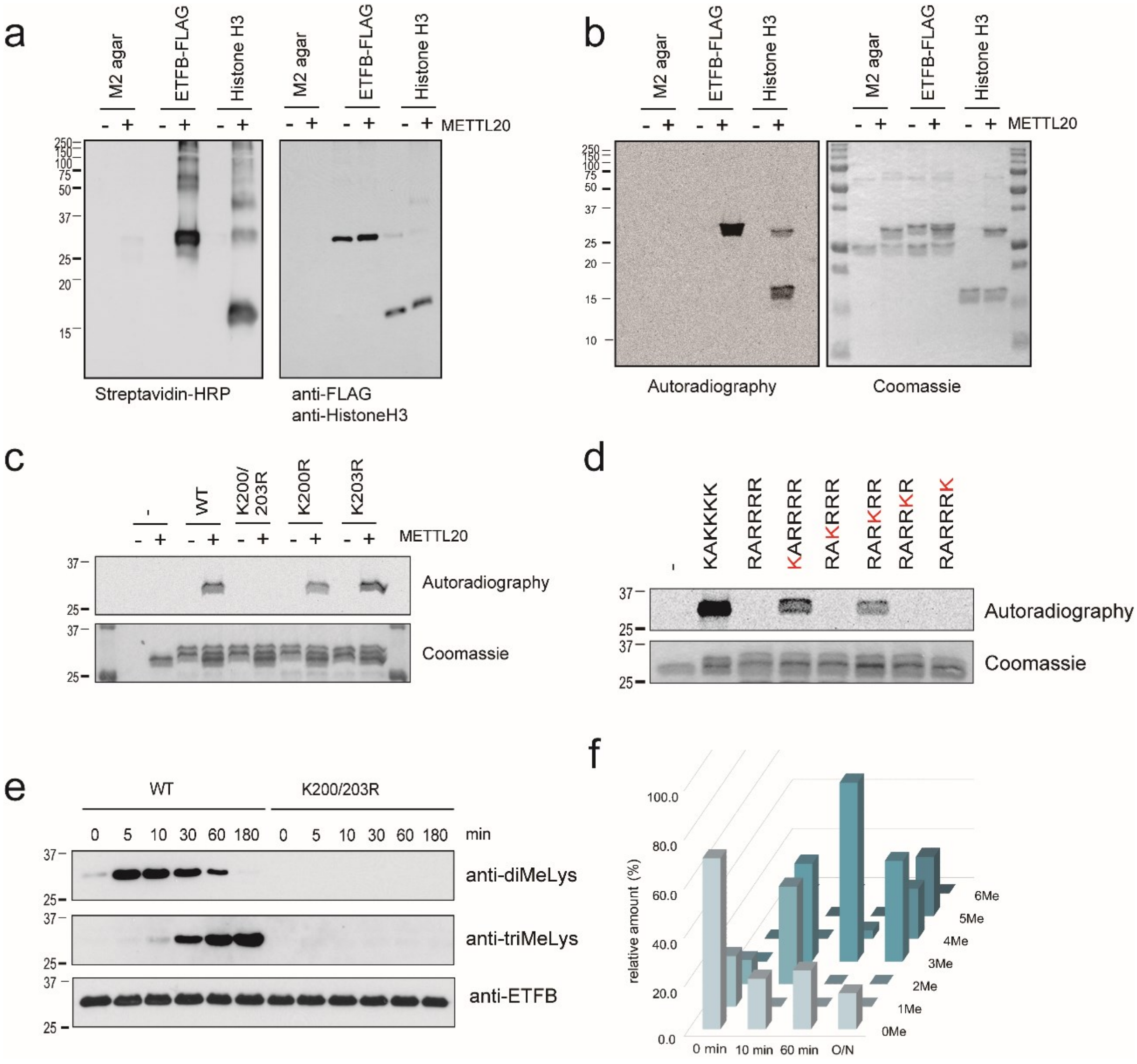

Figure S2. Identification of ETFB as a METTL20 Substrate in mitochondria

(a) FLAG-tagged ETFB or recombinant Histone H3 were incubated with or without His-METTL20 in the presence of ProSeAM for 2 h at 20°C. Modified proteins were biotinylated and detected with streptavidin-HRP (left) or anti-FLAG antibody and anti-Histone H3 antibody for the loading control (right).

(b) FLAG-tagged ETFB were incubated with or without His-METTL20 in the presence of 14C-

labeled AdoMet. Proteins were separated with SDS-PAGE and stained with Coomassie blue (right), the autoradiography was detected with the image analyzer BAS-5000 (left).

(c) Recombinant ETF complex (ETFA WT/ ETFB K200/203R) and His-METTL20 were incubated with 14C-labeled AdoMet for 2 h at 30°C. The autoradiography was detected as in E.

(d) Indicated FLAG-tagged ETFB mutants were incubated with His-METTL20 in the presence of 14C-labeled AdoMet for 2 h at 30°C. The autoradiography was detected as in E.

(e) Recombinant ETF complex and His-METTL20 were incubated with AdoMet for 0 - 180 min at 30°C. The reaction was stopped by adding Laemmli SDS-sample buffer. ETFB were separated with SDS-PAGE, and their methylation were detected with anti-triMeLys antibodies (top), anti-diMeLys antibodies (middle), and anti-ETFB antibodies (bottom, as loading control).

(f) Recombinant ETF complex were incubated with His-METTL20 in the presence of AdoMet for indicated time. After the reaction, ETFB were separated with SDS-PAGE, digested with Asp-N, and methylation of peptides containing K200/203 were analyzed with MALDI-TOF-MS. Relative amount of methylation in ETFB were calculated from the intensity of peptides with 0 to 6 methyl-group.

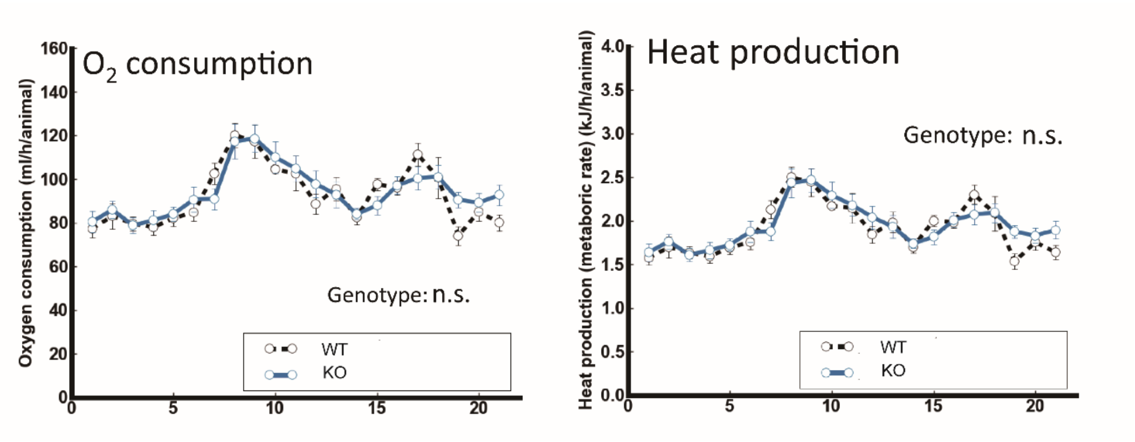

Figure S3. Calorimetric analysis of Mettl20 KO mice under normal diet (ND)

Calorimetric analysis of Mettl20 KO mice fed with ND. ANOVA, effect of genotype, ns; not significant. N = 10 male mice for each genotype at 12 weeks of age.
